# Exploring the analytic process, the analytic third, and the emergence of meaning from a dyadic perspective

**DOI:** 10.3389/fpsyg.2026.1779078

**Published:** 2026-05-25

**Authors:** Anatol Eduard Reghintovschi

**Affiliations:** Faculty of Psychology, Titu Maiorescu University, Bucharest, Romania

**Keywords:** Amalia X, analytic process, analytic third, implicate order, meaning-making

## Abstract

Although the concept of a two-person psychology as an encompassing perspective is not new, the role of the psychoanalytic dyad, or the analytic third, within the analytic process requires further research. Do analytic dyads (or thirds) create meaning and thus contribute to the process? If so, how can this be observed? Assuming the analytic frame is inhabited by the patient, the analyst, and the analytic couple (which includes the third) as the therapy process unfolds, does this member—the analytic dyad, couple, or third—make observable differences within the session? This is the main question. The paper demonstrates, through a model, that dyadic processes involve movements of recursion and growth in complexity. The model is based on the concept of order, starting from similarity and difference. As the dimensions of the psychoanalytic session are considered in a semantic field, producing order is equivalent to producing meaning. Ultimately, it is suggested that the analytic dyad is significant and that, when investigating ‘change’, it follows that the unlocalized ‘participant’ may play a non-negligible role.

## 1 Introduction

As with any original concept, the analytic third (Ogden, 1994; Benjamin, 2004) has a history and many authors who have contributed to its foundation.

The idea of an analytic third involves an unconscious process, an emotional experience, and can be succinctly described as a meaning-making process through sets of intersubjective dynamic patterns that facilitate the emergence of an unlocalized subjectivity.

As with any unconscious process, it must provide inferable manifestations within the session, through its observable counterpart, the analytic couple. Here, the third, as an unlocalized subjectivity, is considered to be contained within the actions of the analytic couple. Within the ongoing movement from “I and you” to “we,” there is a plausible layer of actions from which to infer such an underlying process.

Like any other interrelated unconscious phenomena, such as transference–countertransference as a unitary event (Loewald, 1986, p. 276), the third and its counterpart are hypothesized to be parts of the process. Thus, they are involved in creating new patterns of relating and self-understanding. As with any emerging novelty, or in transforming something unknown into a recognizable entity, this involves the creation of meaning.

Saying that the analytic couple should provide observables requires a tool adequate to the ‘form’ of the analytic process, suitable for observing the patterns generated by the analytic couple. Such patterns (or their absence) indicate the production of order (or noise). As will be shown, these dynamic patterns of interrelation display a continuous oscillation at the level of complexity. In a semantic field (the session), if complexity grows, this is achieved through a continuous production of meaning.

## 2 Analytic couple and tertiary processes

In its beginnings, psychoanalytic observation and understanding established a one-dimensional view of the analytic process. In the well-known search for hidden meanings, everything was ascribed to the patient (or to the therapist’s ‘method’), even if unconscious dialogues (Freud, 1912, p. 115; 1915, p. 194) were recognized early on. We had a one-dimensional system of reference that positioned the analyst as an ‘objective’ observer.

This objectivity assumed that the observer does not influence the observed, and that the act of observation does not change either of them. Yet we deal with *operational concepts* (Loewald, 1971). An operational concept, such as mental space, psychic reality, resistance, repetition, recursion, or transference, serves as an organizing principle, “gradually distilled out of the events when investigated in a certain perspective” (Loewald, 1971). That is, it represents an underlying structure of ideas (or beliefs) regarding *how* something works, which is subject to change.

The recognition of countertransference as an observational tool (Heimann, 1950; Little, 1951; Racker, 1948, 1957) caused the ‘one dimension, one direction’ point of view to fade, making room for the perspective that ‘the therapist matters’ (Luborsky et al., 1997). With the advent of countertransference in the mainstream of theory and technique, a ‘new’ objectivity and a new hermeneutics emerged. This was followed by a new perspective on the process. A “talking cure” involves more than one mind, more than one voice, and the subjects of the couple, of the analytic fields (Baranger and Baranger, 1969/2008, 2009), of the dyads (Stern, 1985; Tronick et al., 1998; Tronick, 2007; Sander, 2008), or of the analytic thirds (Ogden, 1994; Benjamin, 2004) represent new areas of description and conceptualization that imply more than just the analyst and the patient as parts within the analytic process. All these conceptualizations point to something emerging from interaction, something enfolded within interaction, which, in turn, is continuously changing the way the analyst and patient interact.

If we are discussing dyads and dyadic processes (Tronick, 2007, 2013), mutuality and intersubjectivity (Benjamin, 2004, 2016; Sander, 2008), the co-creation and co-production of meaning in the present moment (Stern, 1985, 1995, 2004), and the analytic thirds (Ogden, 1994; Benjamin, 2004), among many other ways of describing something more than the patient and the analyst, then the idea that the analytic couple (or the enfolded analytic third) influences the analytic process becomes plausible.

Mutuality implies “both as one”, while from the couple’s perspective, the couple’s actions involve “one from both”, exhibiting a specific way of evolving. If there is an *autonomous* process involving both analyst and patient in creating an independent unit, questioning what the analytic couple does to its own constituents while being shaped by them (e.g., being with, intersubjective relating, dyadic states, and mutuality) involves many levels of process. Yet mutuality becomes a defining element. As in:

“Understanding would seem to be an act that involves some sort of mutual engagement, a particular form of the meeting of minds. As applied to self-understanding, it would involve the mutual engagement of different mental levels.” (Loewald, 1979, p. 165)

Implicit in the above quote is an interior–exterior understanding of the process, distilled under a “certain perspective,” which is the conceptual core of many theories and methodologies. Our psychoanalytic metaphors are often spatial; “talking of an ‘underworld’ or of depth psychology, we are conceiving psyche on a spatially vertical level” (Buchholz, 2007, p. 164).

Setting ‘communicating’ as a system of reference, “a particular form of the meeting of minds”, or in different words “relating”, is neither inside nor outside. As a semantic field, within “talking to each other” the action involves creating its own domain of existence, thus reapplying “talking to each other.”

What kind of change does such a move imply, from one system of reference to another? Perhaps the most important one is moving away from the risks of circularity and paradox, given the ease with which time as a constituent is eliminated from the basic metaphors we employ in an interior–exterior frame of reference (see infra).

How “objectivity” is conceived equates to determining what ‘observation’ means as doing, or what becomes observation in this or that referential context. As such, in trying to see what and how is happening in a session, one can ask: how can we discern those actions performed by and the patterns made by an “unlocalized subjectivity”?

“Conversation” implies that an utterance is an action^1^, a doing. The dichotomy of hidden/evident is transcended in this view. There is no “geography” recommending maps of meaning. Such “cartography” has no fixed territories, as the environment is built through interaction, thus changing continuously. If observation matters, then the referential is set so “meaning is made” and offers means to answer how and what.

In a dyadic view, there are a patient and an analyst. Considering only bidirectionality, we can devise a linear model that addresses a sequence of actions. The couple is enfolded in what they do, so it can be understood as a non-autonomous process or entity. In a bidimensional or triadic perspective (patient, analyst, and the analytic couple), the analytic couple emerges from their interactions. If it is an autonomous participant, then relating creates a reality. A bidirectional and bidimensional (thus triadic) perspective is necessary for observing a self-directing and self-organizing^2^ entity while creating or dismantling levels of organization—essentially, that which has an “identity.”

## 3 “Similar and different” or/and “inside and outside”

Usually, when an event is observed and reported, the system of reference is implicit and involves both an interior and an exterior. “We frequently conceive of the psyche with metaphors of space” (Buchholz, 2007, p. 164), viewing the “mind is inside”, which establishes a reference under interior and exterior dimensions. Yet any observation made, or any theory devised from it, is highly sensitive to changes occurring within the employed referential, whether in psychoanalysis or elsewhere.

“Order” equates to the outcome, after a certain time, of a (self-organized) system of *relations*. Even if it seems obvious, it should be noted that order (or noise) is not a given; it is achieved and created. In this *system*, where generating “order” is a core factor, “relations” are actions, outcomes of interaction (Enfield, 2013, p. 9), that are

- Enabling the inclusion and retention of all the system’s components, generating *relational content*.
- Creating the conditions for the system to function as a system, generating *relational context*.

An ever-changing environment and the adaptation to it (the session) involve a continuous production of sense and pattern, while failure to adapt produces noise. We can approach an event from either view proposed here; e.g., ‘chaos making’ (order) and ‘boundary violations’ (int. ext.) are ways of describing a series of events.

More order equates to more links, a growth in complexity, and a decrease in entropy, so to speak; i.e., the fabric of relations becomes denser, and, as a main result, new levels may be created through relating.

Starting from here, it is worth asking: why change the referential from the “interior–exterior” metaphor and description to “order” (the production or destruction of it)?

Emergent events invite circularities and sometimes paradoxes when we address an event that involves more than one level, one dimension and one direction, or has “time” as a core factor but is ultimately omitted. Meaning-making is such a process that involves hierarchies and time, as well as various forms of emergence. As a process that influences and changes the very background that generated it, the “interior exterior” or the “geography” of relating, as perspective, is prone to sidestep “time” and “*the fiber of the mind*” (Loewald, 1962), or the creation of new levels and new dimensions of relating out of fluctuations. In “order out of chaos”, employing Prigogine’s expression, order equates to *form, function*, *and fluctuation* (Prigogine, 1997).

I will try to draw a parallel between one perspective and another in a couple of examples that will justify the choice made here of ‘order’ as a provider of a referential.

It should be noted that neither perspective is the “correct” one, nor is one view “better” than the other. A choice is made according to the relationship unfolding between the event, the environment, and the observer involved.

Similar and different are the basic relations that describe “order.” They are elements within a set with only two elements, {s, d} (similar—‘s’, different—‘d’). Chaos could be represented by a complete lack of pattern (as in d, d, d, d, d, …) and perfect order or symmetry is (s, s, s, s, s, …). Everything in between presents configurations of s and d.

We can define “+” as an operation within {s, d}. It establishes “comparing” as a basic operation that eventually informs about the level of order. Implicit and inevitable in this is the contribution of the observer. Thus, ‘+’ functions as a relation between relations. Then “s + s” equates comparing similarity with another relation of similarity, and they cannot be different. If we compare a relation of similarity with one of difference, this cannot be similar, while differences compared to differences cannot be dissimilar; they are similar. We can write the above descriptions of the “+” operation applied to {s, d} as follows: s + s = s; s + d = d; d + s = d; d + d = s. We arrive at a basic mathematical operation called *symmetric difference*^3^.

A different approach is in the case of inside and outside. If “where” and “from where” are questioned (a geometry in the end), then we arrive at a different picture. If “a” and “b” are events set either in here or out there—like in “a thought is an internal event”, or talking is an inside → outside while listening is an outside to inside—we set a “position” and a “boundary.” Under a “from … to…” (an operation noted by an ‘→’), we can draw the *relations* between “a” and “b” as such: (i) int. → int. = int., (ii) int. → ext. = ext., (iii) ext. → int. = int., (iv) ext. → ext. = ext. These equations condense “where” a movement or a link begins (from) and “where” a movement ends (to). Thus, from interior to exterior is an exterior, and vice versa; from exterior to interior, the arrival is an interior. The other two equations are evident.

A process described in terms of the inside–outside domain involves forms and patterns that unfold under introspection and extrospection, seen as complementary “directions.” This includes questioning the meaning-making activities that involve emergent patterns and forms or flattening them into noise.

If *order* exists, then the logic of symmetric difference offers a *binary* view or an answer in s and d. At the same time, considering int. and ext. as descriptors, if emergence is involved, then contradictions and paradoxes could follow.

One example involves ‘spatial’ representations, specifically drawings. Bion, while at the Tavistock on 5 July 1978, describes the following event:

“The child had drawn a picture and told me it was a house and a tree. On the other side of the piece of paper was a meaningless scribble, but if I held that picture up to the light, then the meaningless scribble shone through the paper and the picture was quite different; it was somebody watering a plant.” (Bion, 2014, Vol IX, p. 65)

“Somebody watering a plant” is a *next-level (emergent) event*, neither on one side nor on the other side of the paper. It should be noted that “somebody watering a plant” is also data enfolded within the data.

This brings to the fore a new level of order (a new meaning is created), implicate within looking at one side and then at the other side, and explicate within holding it up to the light. Something ‘meaningless’, a scribble, was transformed into part of a recognizable pattern by considering both sides at the same time.

Considering the above observation in both systems of reference and describing the sequence of events, we can note that setting the spatial metaphor as referential eventually involves an antinomy. Implicit in this sketch of paralleling these systems of reference is the idea that the drawings are communications, ways of conveying meaning through drawings on paper. Thus, we have someone communicating something to someone else, who ultimately observes a *new layer* of this communication (layer is also a ‘spatial’ term).

How can we address, at the level of descriptions, the emergence of the third image when considering a ‘spatial’ perspective? The paper and the drawings take on values within {int., ext., and →} for an observer, where ‘→’ as an operation designates movement from ‘x’ to ‘y’. We can identify one side of the paper as A and the other as B. Regardless of how thin the paper is, A is an ext. to it, and B is an ext. as well. However, A & B is not an ext.; the image results from the overlapping of both drawings: the ‘house and the tree’ or A, and the ‘meaningless scribble’ or B. A&B, or ‘somebody watering a plant’, is situated neither on one side nor the other. Thus, as we have only two values, it is an int.

It makes sense to set the image ‘somebody watering a plant’ as an inside, considering the relationship between the child and the analyst. If we consider the set A, B, and A&B as traces of a process, we can say that the set of drawings is an enfolded definition of containment, if ‘somebody watering a plant’ is about growth.

However, considering the actions in sequence (with A on one side and B on the other, when held in light we observe A&B), we arrive at ext. → ext. = int. This contradicts the basic operations written above. As emergence (or a next-level event) is present, we encounter an antinomy this time. Thus, it does not keep the elements within the system. If A, B, and A&B are viewed as ‘unknowns,’ representing the interior content of the child’s mind unfolding in the form of a drawing, we arrive at int. → int. = int. Hence, if we set aside ‘observation’ and extend the environment under an “operational concept,” a relationship between an unknown meaning enfolded in A and another enfolded in B, which creates A&B, could illuminate what is unfolding (or what is enfolded) between the child and the analyst in their interactions. Perhaps. Yet, circularity is also a potential attribute (or an inevitable outcome) of any conclusion drawn. Thus, we either arrive at an antinomy or at a (potential) circularity.

Next, view: observing A, B, and A&B under “order” as a metaphor. In this context, similarity and difference provide descriptions for what is happening, represented as {s, d, and +}.

We have a couple of drawings and three images. Comparing is the operation defined, noted through ‘+’. We need two drawings to apply the comparison, ‘+’; thus, we need to set a base (one drawing) for applying the operation. First, we keep the first image, A, as a reference element. When comparing the first with itself, the result is an s. Comparing the first with the second image yields a d, and the first image with the third one also results in a d. Regardless of which element is set as the reference for comparison and noting the results—‘observing’ one drawing, ‘house and the tree’, ‘the meaningless scribble,’ and ‘somebody watering a plant’—when describing the steps A, B, and A&B, we arrive at the same equation: s + d = d. It exists within the system.

We do not deal with ‘drawings’ except when working with children and in a few other isolated cases. Thus, drawing should make room for ‘talking to each other’.

Talking, metaphorically, generates both the paper and the drawings. Speaking occupies one side, while listening occupies the other, if communicating is seen as a single event. The main idea is that by keeping speaking and listening in the same ‘light’, we could arrive at “somebody watering a plant” within the fabric of speech.

This *next level event* sketched above should include *how* a conversation event, viewed as an observational tool, functions for observing a conversational event. One such example is found in Thomä’s (1997) description of his *new* understanding of the here and now of transference:

“(…) I had missed a transference interpretation about a day-residue of a dream. Amalia had picked up my idiosyncratic speech acts, which were far from being as clear, distinct and short as an interpretation should be! Quite often I look for the most fitting words, starting anew, changing sentences halfway etc. In Amalia’s dream, a drunken man was stammering and expressed himself in a strange way.” (p. 22)

The understanding of the dream image of a drunken man by Thomä can be seen, when merged with Amalia’s understanding, as “somebody watering a plant”, manifesting through the overlap of both sides of relating and being together. Here, Thomä’s *new* understanding of Amalia’s insights and comments about herself and him equates to “if I held that picture up to the light”, which in this case would be informed by “transference interpreted in the view of plausibility in the here and now”. This represents “a new meaning”, emerging from listening to Amalia now while he reflects on talking to her then. “Plausibility in the here and now” serves as a source for a new metaphor, allowing for the discrimination of *new events in the environment*. Thomä’s understanding of Amalia’s perspective—that “a drunken man was stammering and expressed himself in a strange way”—could be a *next*-*level event* when interpreted by Thomä. Without Thomä’s understanding^4^, Amalia’s oneiric insights shared during the session could not be qualified, as they would remain outside the observable field. Observing entails making distinctions within the observational field of the session, from which meaning is derived. “Somebody watering a plant”, from Thomä’s and Amalia’s perspectives, involves a way of listening to what is said and how it is expressed by both of them. From this, *mutual knowledge* could emerge about how one’s listening and the other’s listening generate a description of what they say, an A&B. Amalia’s understanding *and* Thomä’s understanding, viewed from an interior–exterior perspective, will not permit a finite description of what constitutes ‘mutual knowledge’. A dyadic event is neither “interior” nor “exterior”; it represents “somebody watering a plant” on the fabric of speech, which creates a new level of understanding or delineates a new fact. Thus, delimitating a “fact” is a process that involves (i) an observer who is open and present to, (ii) a corpus of data that includes the observer as well, and (iii) a set of tools for transforming this data, a mapping device, or a metaphor^5^.

In “In Amalia’s dream, a drunken man was stammering and expressed himself in a strange way”, listening under “transference interpreted in the view of plausibility in the here and now” would imply, if *order* is considered, a mirroring of an event (idiosyncratic speech acts, A) by another event (a dream element, B), and yet by another (mutual) event (a realization under a specific way of listening, A&B). This last mirroring, A&B, can be viewed either as an infinite recursion or as *generating* a new level of manifestation. In the following description of “successfully communication”, an “ad infinitum” unfolding is introduced to encompass ‘mutual knowledge’:

“In the process of communication, the ‘sender’s’ communicative intention becomes mutual knowledge to ‘sender’ (S) and ‘receiver’ (H), i.e., S knows that H knows that S knows that H knows (and so ad infinitum) that S has this particular intention. Attaining this state of mutual knowledge of a communicative intention is to have successfully communicated.” (Levinson, 1983, p. 16)

We denote “S knows” with “a” and “H knows” with “b.” So, if [a, b], we deal with an unlimited sequence [a, b, a, b, …]. Alternatively, we deal with a ‘next level event’ that requires a description based on [a, b, a + b]. In this case, “a + b” participates in the description. The term “a + b” denotes a dyadic action, which in this context represents a form of “knowing.” Such a dyadic enterprise operates on a different level, and consequently, “a + b” calls for a new level of description as well (e.g., bidimensional, bidirectional). This new level of description recognizes *the couple as an autonomous participant*, “a + b.”

In the case of mutuality, if successful, we can select an infinite sequence as a description, or propose a new “participant” that integrates everything into one element within a new dimension. The second option opens a workable reality. Thus, the benefits of a triadic view, [a, b, a + b], lie in condensing “and so ad infinitum” into an “a + b” as an autonomous element. The idea is that a pair of elements [a, b] in mutuality gives rise to a triadic base [a, b, a + b] for *describing* the first and second levels of an emerging hierarchy or a new relational reality.

This perspective allows for the observation of new levels of organization as they emerge, levels that could initially be hypothesized as enfolded within the couple’s participation in the analytic process (see infra).

These ideas call for a model. What kind of model? It is worth borrowing the methodological standard from conversation analysis: “observing something and reporting about it in such a fashion that other people can in principle independently check up on it” (Wilson, 2012 p. 219).

## 4 Conversation analysis

Conversation analysis (CA) has been recognized as a valuable tool in psychotherapy research (e.g., Peräkylä, 2008; Peräkylä and Sorjonen, 2012; Buchholz and Kächele, 2013).

Conversation analysis (CA) arose from the fields of sociolinguistics (Goffman, 1964) and ethnomethodology (Garfinkel, 1967) and was initially developed by Sacks (1992) and his colleagues (Sacks et al., 1974) at the University of California in the 1960s and early 1970s (Peräkylä, 2008, p. 12; Buchholz and Kächele, 2013, p. 9). In “A simplest systematics for the organization of turn taking for conversation”, Sacks et al. (1974) assert that turn taking might be a basic form of organization for conversation. In this article, Sacks, Schegloff, and Jefferson presented a model, or set of rules, for a ‘turn-construction component’ and a ‘turn-allocation component’ (pp. 702–703), which together form the system for conversation and open up a research methodology. Wilkinson and Kitzinger (2008) describe that CA methodology involves: “detailed empirical studies of specific, observable, interactional phenomena” and “rests on three fundamental theoretical assumptions: (i) that talk is a form of action; (ii) that action is structurally organized; and (iii) that talk creates and maintains intersubjectivity.” (p. 54).

Employing the analytic material with an inductive mindframe, while placing observation at the core of our inquiry, we come to see that “what psychoanalysis is begins to be defined in terms of what psychoanalysts do. To apply CA methods to psychoanalysis endorses that line of thinking” (Buchholz and Kächele, 2013, p. 6).

As CA does not commit to pre-existing theories and instead adopts an open-minded approach to the exploration of interaction, it uncovers practices and discovers patterns of interaction of which therapists may be unaware (Peräkylä, 2013).

## 5 Building up the model

We can begin with what the analyst and the patient do. “They examine what is talked about and they examine the context in which it is talked about: that is, who they are for each other.” (Levenson, 1983, p. 8).

The psychoanalyst Levenson (1978) asserts that the psychoanalytical process is a language process “which allows for, indeed requires, the synthesis of these two paradoxically oppositional aspects of therapy; the aspect of meaning and the aspect of experience.” (p. 6).

For that synthesis, Levenson proposes an algorithm consisting of three steps:

1. “The establishment and definition of the therapeutic frame.
2. The elaboration and enrichment of implicate and explicate order in the patient’s life.
3. The elucidation of this order in the therapist–patient relationship” (Levenson, 1983, pp. 54–55).

Implicit in Levenson’s algorithm is “the use of language”. The sequence serves as a blueprint for linguistic discourse, encompassing both verbal and non-verbal interaction. Yet implicate and explicate order require a different approach, as this perspective emphasizes the interdependence of relations, replacing the deterministic order of things with an understanding of order that is “neither solely in the subject nor solely in the object, but instead in the cycle of activity that includes both” (Bohm and Peat, 1987, p. 149).

This change of perspective transforms the characteristics of order, defined by succession, from being similar and different to “similar differences and different similarities” (Bohm and Peat, 1987, p. 149) within the elaboration and elucidation seen as actions, and also brings simultaneity into consideration. One can observe this shift in emphasis in Levenson’s idea of the “enactment of the content”, that which suggests “the therapist formulates a truth, and in the process of telling the truth, the therapist participates with the patient around the truth that he is telling the patient” (Levenson, 1978, p. 4).

The concept of “generative order”, which leads to “implicate order” (as used by Levenson), is introduced by David Bohm, one of the most significant physicists of the last century. Generative order involves “similar differences and different similarities”, and within this “order”, the whole does not emerge from the accumulation of details; rather, this kind of order is captured in the process of unfolding *a glimpse of a whole*, emphasizing that “a process of creation may begin from some broad encompassing overall perception” (Bohm and Peat, 1987, p. 160). Implicate order (or enfolded order) is encompassed by the generative order. The essential feature of enfolded order is “the simultaneous presence of sequence of many degrees of enfoldment with similar differences between them” (Bohm and Peat, 1987, p. 174).

Implicate order, sometimes tacitly acknowledged, consists of parts enfolding the whole, contrasting with an “explicate or unfolded order, in which similar differences are all present together, in a manifest and extended form” (Bohm and Peat, 1987, p. 174). It should be noted that in this view, echoing the hermeneutic circle, it is understood that “Language is also an enfolded order. Meaning is enfolded in the structure of the language, and meaning unfolds into thought, feeling, and all the activities” (Bohm and Peat, 1987, p. 185). An example is provided by (Bohm and Peat, 1987, pp. 172–174).

A simple experiment could clarify this concept. It consists of Bohm’s description of a device made of two transparent cylinders, one inside the other, with glycerine filling the space between them. An ink drop is situated in the glycerine, which occupies the interstice between the two glass cylinders. Turning one cylinder causes the ink droplet to become invisible, “lost” in the movements of the ensemble. No order is visible; it is hidden within movement. Reversing the movement restores the ink droplet as if it emerged from nothing. After the first movement, the droplet is *enfolded* into the glycerine (an order while dissipated), and with the reverse movement, the droplet *unfolds* (a different kind of order). The invisible droplet represents the implicate order, while the visible droplet represents the explicate order. This can be likened to “somebody watering a plant”.

In Levenson’s algorithm, the “analytic frame,” once established, serves as the milieu for the manifestation of order through relationships, and “the elaboration and enrichment of implicate and explicate order in the patient’s life” involves how parts enfold the whole and how unfolding allows for the manifestation of a particular event.

Within the algorithm, this dialectic of order is hypothesized to travel from the life of the patient to the life of the analytic couple, creating a (hi)story. As such, the implicate order allows “parts” (or sequences of interaction) with many degrees of enfoldment to evolve, while this hidden order becomes manifest through the unfolding of process in actual interaction, like an ink drop.

As “order” is the current topic of inquiry, i.e., the association of regular and irregular events, of similarities and differences (and “similar differences and different similarities”), their density and form begin to provide an answer.

Observing is defined as making distinctions in one’s environment. Making a distinction within one’s environment, within “order” as referential, belongs not only to the analyst (a) or to the patient (b), but to the analyst, patient, and the analytic couple, to [a, b, a + b], as all are makers of order (or noise).

Observing, seen as an ongoing action within the session, in speaking and listening, becomes a constituent of *how* the system evolves, while the patterns of interaction are enfolding patterns of interaction. Observing is not ‘something’ that happens after the ‘event’ but while the event unfolds; it is part of what is happening. Under observation as action, this involves the emergence of new levels of order as new meanings are created. If the level of order decreases, then observation is either silenced through confusion or replaced by noise-making actions (e.g., attacks on linking).

Considering again the episode “a drunken man was stammering,” if *order* is considered, we deal with a mirroring of an event (idiosyncratic speech acts, A) by another event (a dream element, B). Order is already undergoing a “less → more” transition, as the dream element involves a similarity present in the here and now. Order undergoes another “less → more” transition through a “realization under a specific way of listening” that can potentially build an A&B, a state of mutual understanding. With mutuality established, with A&B, there is a new level of manifestation emerging from A and B. The sequence A → B → A&B involves, within a “less → more” transition at the level of order, the emergence of a new *dimension* in relating, described as ‘mutuality’.

So, what qualifies as “making distinctions in [a, b, a + b]‘s environment”? The form that observing takes in [a, b, a + b] implies distinctions in [a, b, a + b]‘s environment (the session is seen as a process) and the creation of links between distinctions, e.g., mirroring, that ultimately involves the ongoing production of meaning. This description implies that mutuality, or a + b, emerges from the ongoing process of meaning-making.

Even if this idea seems to be confined by circularity, we should remember that “a + b” represents a new emergent level, as in the event of “somebody watering a plant,” which allows us to relate processes in a new dimension, integrating within the linearity of “a then b then a then…” the bi-dimensionality of “a + b.”

Thus, observing becomes the act of *making distinctions and links* within the environment of [a, b, a + b]. Observing unfolds within similarities (enfolding links) and differences (enfolding distinctions) and level stepping, all seen as parts of the session and as processes.

In a semantic field, we can perform a similar “questioning” regarding the content of a pair of verbal interaction events, e.g., CA. This meaning and that meaning—are they similar or different?

The reason for comparing this event with that event (e.g., an utterance, a narrative) in terms of content within a specific context lies in approaching a complex system (the session) regarding the level of “order.” If the system evolves from less to more order, then there is an inherent production of meaning. In exploring how “order” progresses, unfolding a production of meaning (or noise), we shift the focus towards relations between relations or relations embedding relations.

**Figure 1:**
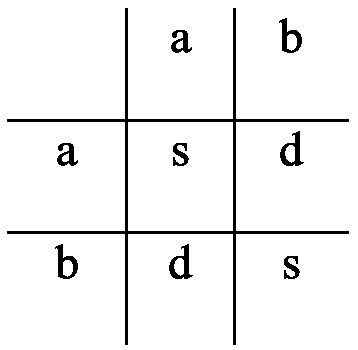
Discrimination as operation.

Similar and different are the basic relations that describe “order,” or in other words, the basic operation is discrimination. Everything starts from a single binary process called “discrimination” (Bastin et al., 1979, p.455). Discrimination, by definition, is the elementary act or operation of comparing one autonomous element with another under the question, “Are they similar or different?” (Figure 1, where label ‘s’ denotes similar and ‘d’ denotes different). The result consists of two different strings (the first element “a” and the second “b” coalesced by the observer through discrimination). Autonomous elements stand out from a background through their internal interconnectedness (see Varela, 1988, p.214).

A note regarding “similar.” Here, “similar” and “functionally equivalent” are interchangeable descriptions. Functionally equivalent refers to exchanges such as “hello” and the response “good morning.” Even if they “differ” in wording, they are functionally equivalent, executing the same conversational function, i.e., “greeting.” In this case, “Are these functionally equivalent or different?” provides a mapping, applying “discrimination” as an operation onto the group of sentences and advancing towards “similar differences and different similarities”. It emphasizes the “function” of what something does in the process of comparison. The opposite side, where the same statement serves various functions, is also important. For example, “Is this your pen?” may be asked by someone who needs it to sign a paper, seeking permission to use it, which is different from the same question a mother asks to prompt her child to pick up the pen from the ground. Similarly, a “good morning” in the middle of the day may equate to a “wake up” rather than a “greeting,” addressing a shift from sleep to what is happening.

In a verbal interaction event, there are numerous interdependent semantic layers. Shared meaning, viewed as a relational achievement, transforms a verbal exchange into dialogue (Bohm, 1996/2004, p. 3), or ‘items of information’ through understanding into a “meeting of minds” (Loewald, 1980, p. 382). Shared meaning involves more than token correspondence; it encompasses mutual understanding and the co-creation of content and context. Accordingly, conveying (or imposing) opinions and dialogue are on opposite sides of relating. If shared meaning is at work, then what is ‘shared’ emerges through mutual understanding (implicate order) and generates its own domain of reapplication (explicate order). Francisco Varela’s (1988) metaphor for describing enactive cognition, “laying down a path in walking”, illustrates the unfolding of autonomous elements as they generate their own domain of reapplication.

In a field of dialogue, an utterance grows from an understanding, involving a transition from the local coherence of interaction (explicate order) to the overall cohesion of talk (implicate order) and vice versa.

For ‘B’ to be *functionally equivalent* to ‘A’, a particular ‘meaning’ needs to be reappraised and developed. Determining if one autonomous element within one strand of talk is functionally equivalent to another autonomous element involves several steps. The first step is identifying the autonomous elements (sentences, narratives, etc.). The second step involves determining what episode or what larger unit they create (e.g., a dream telling and a narrative). Episodes have an identifiable beginning and an identifiable end. An episode, as a larger unit of the session, like a sentence, stands out from the background (the session as a unit) through its own internal interconnectedness. The episode provides the first level of context for each autonomous element occurring within it; they create larger contexts through interaction, while the final level is identified with the entire session.

With these two steps completed, the operation of discrimination, “are they similar or different?”, can be applied. Finding an “A → A” (repetition, circularity) or an “A → AX” (recursion, progression) indicates similarity (all other instances are differences).

Establishing B as a functional equivalent of A involves (i) comparing the meaning of each autonomous element in its own context, (ii) comparing the functions they perform, and (iii) establishing whether the content and action of A correspond with the content and action of B. If such a correspondence is visible, then they qualify for a relation of equivalence.

The elements are relations that follow the actual relations in how they unfold. In fact, what is preserved through this model is similarity and difference as relations in their concatenation, and how complexity grows or declines in the session, which is an explicate order.

Considering Loewald’s (1986) observation, it is “impossible, to treat transference and countertransference as separate issues.” (p. 276); thus, a relational event, i.e., jointly created, enfolds layers of *non-separate events*.

“They talk to each other”, paraphrasing Freud (1959/1926, vol. XX, p. 187), provides the environment, i.e., a verbal and an extra-verbal milieu, and the act of talking inscribes the distinctions in the environment. It is necessary to consider a new form of organization, as both a required condition and an outcome. A new dimension, as the environment enfolds the very act of “talking to each other”. If transference *and* countertransference form a unitary event, mirroring this idea implies that “speaking *and* listening” build a unit, considering that utterances “make meaning as they unfold, not after they are finished” (Brazil, 1995 p. 17).

Conversation is a surface event. The description “The stuff a clinical situation is made of is conversation” (Buchholz, 2019, p. 87) involves more than succession; in this description, simultaneity plays a role as well. Making a distinction in such an *environment* implies forms inscribed by the analytic couple in the fabric of language use. There are no meaningful actions in a relational void. What qualifies as “making a distinction” involves similarities and differences (and “similar differences and different similarities”), created by how the environment is changed.

What differentiates the consulting room from other life circumstances? The answer lies in the context that makes possible the events in the form we call analysis. That is the analytic frame, how “observing” (in speaking and listening) emerges. There are countless contents and relations between them; there are numerous relational nuances and shapes, all requiring a foundation, stability for fostering variance, and eventually qualitative change. Within the sequence “frame” → “elaboration and enrichment” → “enacting of content” → “next-level-event,” the frame establishes the first level of context. Rules create the roles.

Until now, the analytic frame, as part of the algorithm, has been set aside, but this complex assembly of guides and rules of conduct plays its part. For example, when speaking of intersubjectivity, the “rules” are exactly what “enable an intersubjective exchange to take place” (Thomä and Kächele, 1987, p.217). If “intersubjectivity,” “mutuality,” or “joint attention” matters, then the context within which theories of observation (how distinctions are drawn and links made to the environment) and observation of theories (what distinctions and links) meet requires “rules,” as rules “create an identity of meaning” (Thomä and Kächele, 1987, p.216).

Considering only the meeting of two practices, namely “free-association (↔ free-floating attention)” and “neutrality,” if a triadic point of view is taken, then what occupies the foreground is free association (↔ free-floating attention) *and* neutrality, merging into a unitary process.

The following model will be applied to one session (the 98th) from the Amalia^6^ X case. Similar/different and successive/simultaneous are pairs of complementary relations between elements that can describe the session; they offer the architecture of the “map.” An utterance, as a unit, is seen as having one side related to the speech part of interaction and the other side related to the listening part. By combining them into a single event and noting it as such, we arrive at a description of *one* event, as performed by both, by the analytic couple.

An utterance, unlike a word, is endowed with autonomy, even if it is multi-dimensional and acquires new meanings with any slight change of context. Autonomy makes a difference. Auer (2015) designates a sentence as “a self-contained linguistic unit” (p. 27). A sentence or an utterance, when considering speaking-listening, has a beginning and an end, which qualifies it as a suitable autonomous or self-contained parameter. An utterance is a co-authored variable of the session and provides a good segmentation criterion. An utterance is also an *observable* element.

## 6 Completing and applying the model

Everything begins with a question and develops according to a specific operation. A sequence of sentences becomes a story (or disorganizes and becomes semantic noise), following the pattern of one event feeding another (larger) event (e.g., phonemes → words → sentences → narratives → couple’s narratives → …).

**Figure 2:**
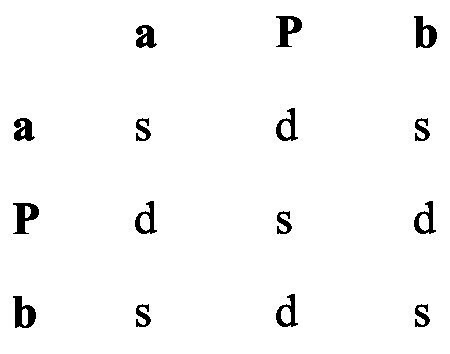
A single pair of sentences.

In terms of quantification, the session could provide the units if examined in isolation, while everything relates to everything else, following Bohm’s idea of order. The autonomous elements (a sentence, a narrative) are the session’s heartbeats. They provide the elements that build up the matrices, mirroring the session onto itself. For example, a single pair of sentences from Amalia X, 98^th^ session:

- *P: I keep on dreaming of my mother, (2.s)*
- *this woman appears again and again.*
  The first sentence (a) and the second sentence (b) are *functionally equivalent*; thus, we encounter a symmetry in terms of content. This operation keeps content and context together and promotes a bi-dimensional perspective, as in the speech–listening pair. For each “similarity” the elements are coded with “s,” and for difference, we code with “d.”
  So, representing the first and second sentences in the sequence, we have: a, P (2s), and b. The next step is to consider a and b in both dimensions while maintaining the operation. This relates to the other complementary pair of relations: “a” is present in what is spoken and, at the same time, in what is listened to. On the horizontal (Figure 2), we have the first dimension of what is said, including the pause— a, P, b; on the vertical, we have what is listened to— a, P, b (P is the pause).
  Discrimination generates a sequence of labels (s denotes similar/d denotes different), while the matrix is built considering that speech and listening form a single event. CA informs the answers. The labels describe the relationship between one utterance and another in speaking–listening. We arrive at a 3 × 3 matrix that constitutes an arrangement of elements following the original source. Following the session, we can see *how* order is acquired while new levels of interaction emerge from recursion. Labels are signposts indicating *how* interaction unfolds.
*First movement*

1. *T: I was delayed by a phone call,*
2. *can you stay a little longer?*
3. *P: I can, yes.*
4. *T: yes.*

**Figure 3:**
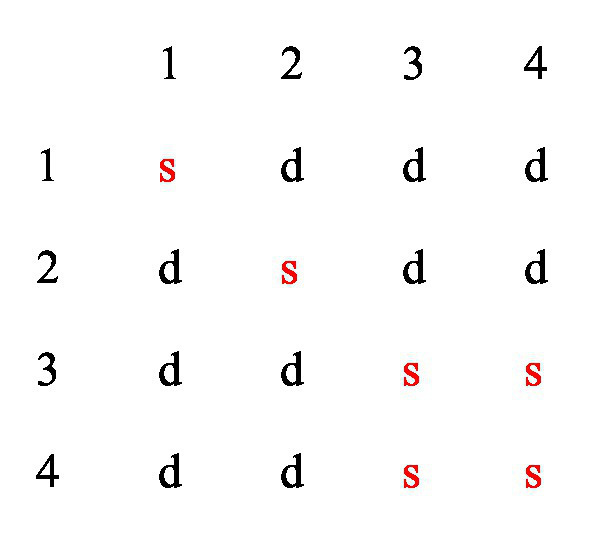
First movement.

The session begins with a negotiation regarding the timing. Analyst and patient agree on shifting the session time. Within the labeling, ‘s’ signifies functionally equivalent (*similarity*) and ‘d’ signifies *difference*:

In this format, what is under the diagonal represents *potential* (Figure 3), while what is above constructs the *history* of this interaction.

The first position in the matrix (line 1 × column 1) is noted with (s) as it relates the sentence to itself. The second position (line 1 × column 2) indicates that the content of the first utterance (1) is different from the content of the second utterance (2); they also perform different functions: one informs, the other requests, therefore it is noted with ‘d’. In the case of ‘3’ and ‘4’, the labels are s’s, as they both agree and contribute to *closing* this piece of interaction. As such, ‘3’ and ‘4’ are considered functionally equivalent.

Why does the pair formed by “3”—“I can, yes” and “4”—“Yes” qualify for functional equivalence? Amalia’s answer closes the request, i.e., Thomä’s question/request is answered, and the second “yes” acknowledges the agreement while closing the ‘deal’, a second closing. One “yes” affirms the deal, and Amalia acknowledges and confirms it. The second “yes” affirms the first yes regarding the deal, providing acknowledgment and confirmation. So, is this second ‘yes’ different from the first one? In terms of function, it is not; they are equivalent.

Amalia’s short answer could be interpreted in other ways. If she asks for her time, for the floor, then ‘yes’—‘yes’ is an agreement in terms of the rules of the game, i.e., of the frame (as in Levenson’s algorithm).

It is the same kind of ‘s’ that results in pairing—“this is mine” followed by “yes, this is yours”. But again, there is an equivalence. A ‘closed deal’ as an action performed has confirmation in line ‘5’ below (it has other functions as well), where it is also announced that something is ‘discharged’.

*Second movement*

1. *P: today there’s no cousin coming.*
2. *(31.0) P_1_*
3. *Well, I keep on dreaming of my mother,*
4. *(2.0) P_2_*
5. *this woman appears again, and again*
6. *(9.5) P_3_*
7. *she is always there like my shadow*
8. *(2.0) P_4_*
9. *or like my tray or*
10. *(--) I don’t know what.*
11. *(3.0) P_5_*
12. *(P sighs)*
13. *(33.0) P_6_*
14. *P: and the other day (-) on the dream with the snakes,*
15. *P_7_ (1.5)*
16. *I said, there is one piece missing. (--)*
17. *T: mh.*
18. *P: very peculiar, the piece was (-) actually the one that I could remember best the next morning*
19. *T: mh.*
20. *P: I remember it now*
21. *and that it came into my mind at midday,*
22. *after I had been here with you, (-) again.*
23. *(2.0) P_8_*
24. *T: mh.*

Labeling the fragment as described above, we arrive at a matrix representing this piece of interaction, i.e., a ‘*sentence score*’.

**Figure 4:**
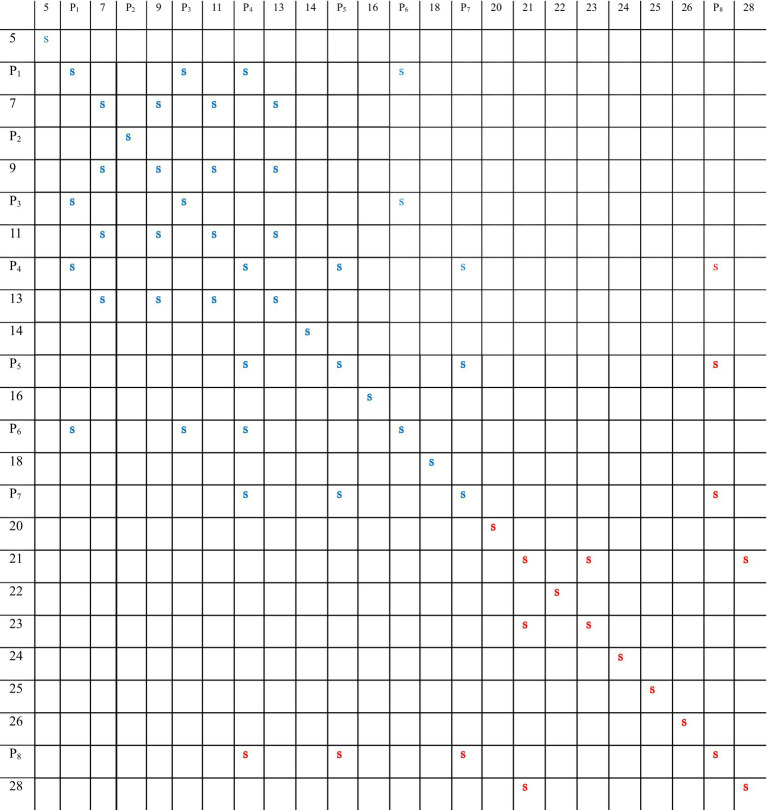
Second movement as matrix of labels (all empty squares are d’s).

For comparing this matrix with the following ones, the empty squares from Figure 4 (and the following ones) designate ‘d’s’. The reason for omitting the d’s is that comparing this matrix with the following ones makes it easier to notice the similarities of form. The diagonal, where the elements ‘meet’ with themselves, is a ‘timeline’ (in succession simultaneity).

In the horizontal (the sequence of spoken utterances), we have the sequence of autonomous elements 5, P_1_, 7, P_2_, 9, P_3_, 10, … as they follow in the session. In the vertical (which represents the elements in simultaneity, listened), we have the same events, in the same order as on the horizontal: 5, P_1_, 7, P_2_, 9, P_3_, 11, …. As we move away from the diagonal, each square represents a relation (the matrix is recording all, each element relating with every other autonomous element from the movement), a position where an element is compared with another element (are they functionally equivalent or not?) and the relation in between is qualified and inscribed accordingly (s/d).

The red/blue s’s are the parts that, in the development of the session, enter recursion. The left corner (blue) has several pairs labelled with “s.” In the table, we note with ‘s’ the squares corresponding to line ‘5’ and column ‘7’ or line ‘7’ and column ‘5’. Thus, in the matrix, there are ‘5’ and ‘7’: Well, I keep on dreaming of my mother, / this woman appears again and again; and ‘9’ and ‘11’: she is always there, like my shadow / or like my tray or…; and ‘20’, ‘22’, and ‘27’—analyst’s “mh.”

Why do ‘5’ and ‘7’ qualify as functionally equivalent? In terms of actions, Amalia reports something, and this something has a content that refers to ‘mother’. Both are ‘reports about’, and their content refers to ‘mother’. The content is reiterated, and through reiteration, it is presented by demonstrating how the content is conveyed. She speaks about an ‘again and again’ and is reiterating it, creating an ‘again and again’ from it. This is a subtle mirroring of what is conveyed through how it is conveyed.

By performing a reiterated report, Amalia gathers a new meaning regarding this “again and again” of ‘mother’. As we will see, this is a procedure eventually performed by both that arrives, step by step, at encompassing larger and larger parts.

It is a complaint regarding a previous ‘time’ (or a previous ‘moment’ of the session), which, through reiteration, moves it from ‘then’ into a “here and now”. An ‘again and again’ happens now, by establishing “how” she reports. Or we can say it is a transference–countertransference movement. As such, there is something regarding Amalia’s mother (from the dream) that equates to something from Thomä (from the session). Speculating, considering that the reverse of a ‘too much of a presence’ is an absence, through reiterating—by creating an ‘again and again’ about an ‘again and again’—she complains about (i) ‘too much of mother’ and, at the same time, about (ii) an ‘absence’, a ‘no Thomä’ (the previous phone call).

At the beginning of the fragment, she is announcing an “absence” (the cousin, line 5), and subsequently, Amalia comments on it by ‘how’ she complains about a “too intrusive mother”. Returning to equivalences and labeling in ‘5’ and ‘7’ (as well as in lines ‘9’ and ‘11’), Amalia reports, and reports about ‘mother’. What she does (both are reporting about or/and complaining about) and the content of her utterances (both are about mother) qualifies the pair of autonomous elements as functionally equivalent; thus, they acquire an ‘s’ through comparison.

The procedure employed above is (i) identifying the ‘function’ performed; (ii) searching whether, in the specific context, the content is or becomes common or not (e.g., mother, woman, shadow, tray, I do not know—are coalesced within a single semantic domain); (iii) comparing this function and content of this autonomous element with the function and content of that element (i.e., are they functionally equivalent?).

If function (f) and content (c) overlap, then we can note the pair under equivalence through discrimination. Moving further in the fragment, other elements require elucidation: how are they employed in representing the session within these matrices?

Within such wordless events, meaning is conveyed, created, or altered, while changing the context ‘creates new events through not speaking’. A moment of silence is an interpersonal occurrence. Bonacchi (2021), from a linguistic perspective (CA), distinguishes between “*dialogical* situations, in which our communicative behavior (verbal and nonverbal) is Other-referential, i.e., directed to the Other, and *non-dialogical*, i.e., concerning self-referential situations in which we do not want to reach the Other.”

Considering P_1_ (31 s) and P_2_ (2 s), we can say that they are different. But how? Bonacchi’s criterion helps begin to distinguish between P_1_ (31 s) and P_2_ (2 s), noting that “the difference between silence states that emerge in situations in which speech, i.e., interactional exchange through words, is considered not necessary (*non-dialogical* situations, NDSs) and silence states that emerge when speech is expected for the communicative exchange with the Addressee (*dialogical* interactions or situations—DS) (Bonacchi, 2021, p.42).”

According to this criterion, the difference can be summarized as follows: P_1_ performs a break, at the level of interaction; in 30 s, Amalia and Thomä’s way of relating changes from talking to not talking, from interacting through speech to interacting through silence. It is an NDS, as interaction through words is made apparently “not necessary.” In contrast, P_2_ performs a continuation; it is not a break in interaction, qualifying as a DS. The first and third pauses achieve a similar action; they are interactions through silence, again labeled as functionally equivalent and different from the second pause.

We can also consider the *content* of these silences as actions (e.g., P_1_, P_3_ compared with P_2_, P_5_, or P_7_), mostly from a clinical perspective, as we arrive, like Dr. Thomä, at the position where we have “to have a high level of inferential effort in the reconstruction of *what is not said but meant*, and *why what is meant is not said*” (Bonacchi, 2021, p. 51).

The pauses seem to fit into two types of actions: the first is a break in interaction (NDS), i.e., communicating a ‘separation from’ (e.g., P_1_ 31s), which is a movement from words to silence, while the second is a break in speech (e.g., P_2_ 2s), allowing the speech to continue. The first occurs in *relating* with the analyst, while the second pause occurs in *talking* while producing an idea, a report, a complaint, etc., preserving the relationship (DS). P_1_ generates a distancing, acting on the relationship (NDS).

*What* is said also concerns a rejection, i.e., setting a distance from the upsetting object. The *context* created by the form of speech, which is *what* Amalia does by telling and *how* she tells (she is fed up with her mother), through breaking her relationship with her analyst, seems to conjoin a double rejection and an ‘enacting of content’. A ‘no’ (rejecting mother) is accompanied by another ‘no’ (silence as distance, NDS). Amalia reports something; she is putting distance from it (the untold dream). This ‘distancing’ becomes installed in the relationship through an event, getting ‘enacted’ by a break in relating. What and how Amalia reports aim at the same end, i.e., generating distancing.

We can see in this sequence that if silences occupy the foreground, i.e., as performed events, there is a reiteration of mirroring. There is an action (e.g., reporting and rejecting, thus installing distance) achieved in a specific manner (i.e., long silences creating distance between them). Content is mirrored by an action, i.e., by how the context is modulated. There is an ‘again and again’ that builds up a negative (mother, this woman, shadow, tray, I do not know); with each step, there is a devaluation of the qualifying mother (i.e., this woman = shadow = tray) to acquire “distancing,” and an ‘again and again’ in the sequence of silences, performing ‘acquiring distance’ within the relationship with Thomä. There is even an ‘again and again’ linking “setting aside” from line 5 (the cousin) with line 20, “forgetting” (the forgotten dream piece), both installing a “distance” between subject and object. In terms of content, there is a at ‘no’ the beginning of the sequence, and afterward, a reiteration of rejection (in line 20, there is ‘something missing’ that emerges following a long pause, which is a reversal of the beginning when an absence in speech follows the reporting that someone is set aside, forgotten).

Comparing this fragment and the next one (the dream telling), one can ask: is this the beginning of a recursion through all these instances of mirroring? Is the “dream” manifesting before “telling the dream”?

*Third movement (first dream)*

**Figure 5:**
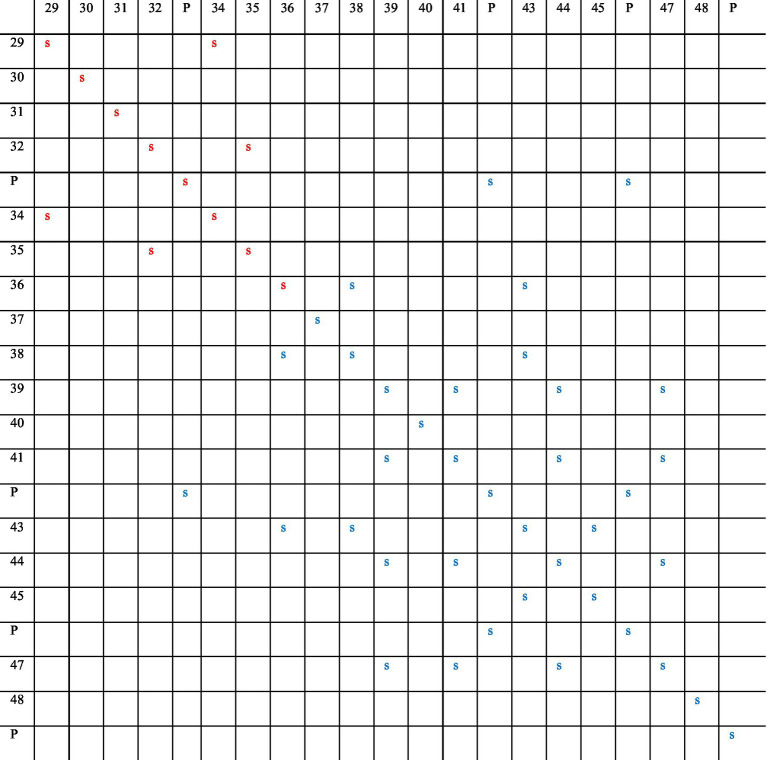
Third movement, first dream.

**Figure 6:**
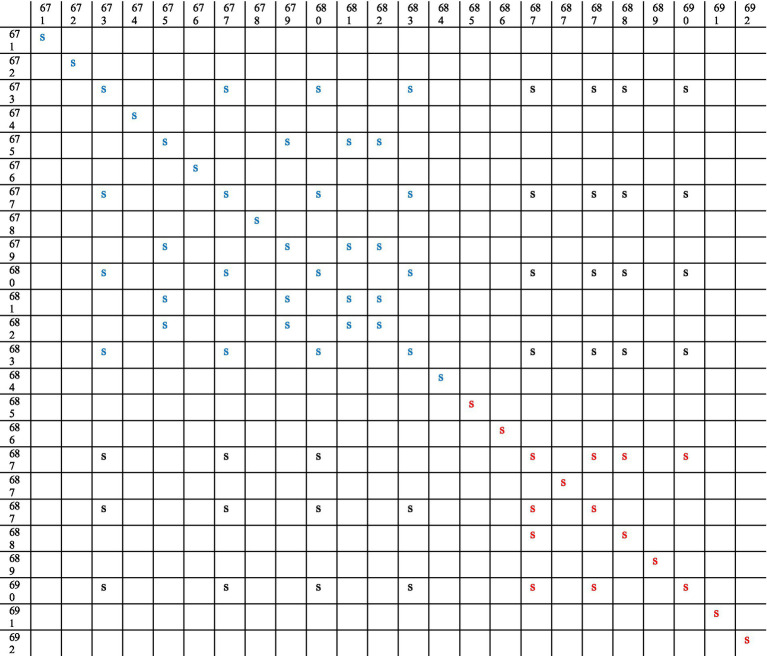
Snow white sequence.

1. *P: namely, I don’t know any more when exactly,*
2. *I just think before this snake story (-) during the exam scene*
3. *(--) I stood there*
4. *and, and I had a completely wet head (--) and*
5. *(1.5)*
6. *I don’t know,*
7. *whether I (-) whether I, uh, had washed hair.*
8. *anyway, (-) I had at the back (-) very huge spots as,*
9. *it wasn’t like shaved but (.)*
10. *somehow there were very huge holes in the hair*
11. *it was so terrible,*
12. *and I stood in front of the mirror*
13. *and looked at them and was (.) really horrified.*
14. *(1.8)*
15. *enormous (.) wholes and then these wet strands,*
16. *it was awful. (-)*
17. *this was the piece, that slipped my memory.*
18. *(2.5)*
19. *that actually terrified me,*
20. *like I said, most (-) the next morning.*
21. *(7.0)*
  In the matrix below, the s’ occurrences are within a single strand of action—Amalia is recounting a forgotten dream. From the 29th to the 49th line, there is one action, namely, she performs a dream-telling. The presence of an ‘s’ here and an ‘s’ there reflects the content of each autonomous element, as each autonomous unit belongs to the same strand of actions. Thus, the first pair, i.e., lines 29 and 34, qualifies for equivalence, given by “I don’t know”. Lines 32 and 35 equate having a “wet head” with having “washed hair”. Lines 36, 38, and 43 address “huge holes in the hair”, while the sequence formed by lines 39, 41, 44, and 47 equates, under overlapping meanings, “terrible” with “horrified,” “awful,” and “terrified”.
  Within “Amalia recounts a dream,” the recurrence of content establishes equivalences. Each ‘s’ is counted based on the previous procedure: (i) identifying the ‘function’ performed (here, dream telling); (ii) searching for common contents in the specific context; and (iii) comparing this autonomous element with that autonomous element (are they functionally equivalent?).
  We have arrived at a distribution of s’s within the matrix, a “form”. The configuration obtained points to the ‘form’ achieved in the second movement. The correspondence between the matrix from Figure 4 and the matrix from Figure 5 shows a complex movement achieved by their interaction, i.e., by the analytic couple.
  The distribution of functional equivalent pairs from this part of the session mirrors, in reverse, the distribution of functional equivalent pairs from the previous segment. Thus, this distribution, while constituting a specific pattern, points to the previous pattern. This reflection generates more structure (i.e., more links and more symmetry). One assumption is that the “form” recurs as soon as the “dream” recurs.
  The forms of the matrices of the second and third movements could be qualified as a recursion. The blue & red/red & blue elements from Figures 5, 6 compared show a reversed recursion. An A[B[C]] → C[B[A]] and it is the recursive movement that they build together.
  A different recursion can be seen in that a forgotten dream is initially shared through silences; a forgotten event shared through an absence of words. In the score of the dream, the ‘story’ is told by what is said, and the ‘story’ is also conveyed by the lack of speech (P_2_ → P_6_).
  The content of the dream is also remarkable, in terms of conveying something through an absence of telling. In what way? A subtle movement seems to emerge; we deal with an unsaid that preannounces an image that leaves an impression on the dreamer through what is not there, e.g., “somehow there were very huge holes in the hair”.
  Cannot be random that such “image” is first forgotten, then conveyed (initially) through how Amalia is not talking, through the huge gaps in the fabric of words. And, on top of this sequence of events, we should note the issue of hirsutism that made such a mark on Amalia.
  Returning to form, the isomorphism underlined above shows that the movement inscribed in one and the movement inscribed in the second, when compared, confirm that recursion is at work.
  The previous idea that Amalia ‘tells’ the dream through silences, before expressing the dream in words, is supported by this recurrence of the distribution of s’s within the matrix. We encountered a recurrent pattern that leads us to assume an ordered kind of interaction that could belong to the analytic couple as a silent participant in the shaping of interaction.
  Is this recurrence of form a way the *analytic couple* organizes ‘an experience’? Or a way of “elaboration and enrichment” enfolded in the analytic couple working through the interactional event represented by the dream?
  The dream comes to the foreground again in the second part of the session. This time, the dream narrative follows a moment of misunderstanding. Amalia clarifies to the analyst which dream she is referring to and when. She proceeds to recount the dream. In the first sequence, the dream was foreshadowed by how she was talking about her mother. The ‘wholes’ in the speech mirrored the wholes occurring later in the dream. Now, in this recurrence of the dream, she integrates ‘wholes’ (pauses) and the dream narrative into one single piece of verbal interaction. The result is a new recursion. Here is the fragment:
22. *large tufts or (-) wholes or (-)*
23. *let’s put it that way. in any case*
24. *(1.2) (P1)*
25. *I was in my apartment*
26. *and stood in front of the mirror*
27. *and (--) I just did,*
28. *(1.5) (P2)*
29. *I don’t know, (-)*
30. *in principal you need a second mirror for (-) looking behind, but*
31. *(1.8) (P3)*
32. *it was just one mirror to look in,*
33. *only one mirror.*
34. *(4.0) (P4)*
35. *and the peculiar,*
36. *when I am thinking about it now, is, (--)*
37. *that I (-) actually saw just me from behind in the mirror*
38. *(--) although I stood, (2.0) (P5) openly (1.5) (P6) in front of it.*
39. *(4.5) (P7)*
40. *I don’t know whether mirrors have a meaning (P laughs).*
41. *(1.5) (P8)*
42. *well, mirror, mirror on the wall, uh-*
43. *yes. (-) sure.*

With Figure 6, a new “A → AX” manifests, adding to the previous recursion in an ABC → CBA → ABC. In terms of form (which also addresses the dream, thus content plays a role as well), this sequence mirrors the one from the third movement, the dream telling (thus it is a reverse mirroring of the second movement). The form obtained after identifying what is functionally equivalent in the narrative mirrors the first couple of forms, with several (minor) differences.

There are several pairs that build the form in Figure 6, such as 671, 681, and 682 (the function is reporting the dream, while the content is I stood in front of a mirror) that qualify as functionally equivalent. Additionally, the pauses in speech, which here, as in the first fragment, create a specific pattern.

They are also considered equivalent in the sequence. Line 287 is split into three parts, all noted with ‘287,’ as we have one utterance and two pauses splitting it; thus, the line needs to be represented in more than one ‘raw’ × ‘column’.

The interpretation given by the analyst—“mirror, mirror on the wall—addresses both moments questioned here: in the first movement, Amalia wants to evade and escape her mother, as well as the dream itself, a second movement, which together build a Snow White theme that is completed with Thomä’s interpretation.

In terms of content, we can find even more. If the first piece (impressions about Amalia’s mother) is revisited in the second one (the dream), then “mirror, mirror on the wall” qualifies as an *interpretation* referring to the classic fairy tale of jealousy and envy, both in relation to the dream and to the present moment of the session. Here, in this subtle import made by Thomä, these aspects of “I want to get rid of her” open the sequence: “mirror → hair issues → negative feelings” or “mirror → hair issues → envy/jealousy”, if we consider Thomä’s interpretation. A meaning is *jointly* achieved.

Within “mirror → hair issues → envy/jealousy”, both the second movement, when Amalia distances herself from “mother, woman, shadow, tray”, and the third fragment, when the consulted mirror effects a new distancing, are condensed. Amalia feels “*terrible, horrified, awful and terrified*” (a sequence that equates mother with mirror, through performing the same pattern of ‘negative feelings generate distancing’). The sequence formed by assembling these movements under recursion coalesces into a single idea, as expressed by Thomä, “mirror, mirror on the wall”. Notably, Amalia’s reaction in 692 (yes, sure) represents an abrupt distancing.

Still reflecting on the relationship between episodes, i.e., how they concatenate and what such a sequence builds, it can be seen that the equation “mother → distance”, assembled in the second movement, is followed by, in the dream-telling episode, “negative feelings → distance” (Amalia distances herself first by forgetting this part of the dream and then by shaping the sequence—terrible, horrified, awful, and terrified; she distances herself again). Afterward, in the Snow White episode, it becomes “mother = negative feelings”, which shows another aspect supported by the current model.

The sequence of episodes functions as an “emotional syllogism,” so to speak, which is built up by the couple. This third ‘equation’ adds a new semantic layer to “mother”, or to “mother = analyst” (i.e., to the current transference–countertransference movements), enriching the understanding of the events of the session with new potential meanings regarding what they achieve, i.e., what the analytic couple accomplishes in overcoming the relational heritage that Amalia possessed, which shaped her life.

Recursion, or mirroring, seems to be part of a more complex process that self-organizes the analytic couple’s actions. Comparing the current matrix (Figure 6) with the previous ones (Figures 4, 5), in all the “dream,” a character plays a leading part; there is also an equivalence at work in terms of form (arrangements of ‘s’s’) in these matrices.

With this last step in the sequence, we can note that in this session, whenever the dream is a subject that offers content for their interaction, the equivalent autonomous elements get organized into a recurring form.

This correspondence between “content” and “form”, which manifests through how Amalia and Thomä interact, is far from random due to its complexity and precision. Recursion, in this case, feeds the transformation. This new observation—that when “the forgotten dream” surfaces in interaction, an equivalent form emerges—is remarkable regarding *how* structure complexifies.

The path that links the forgotten dream manifested through what is *not* said with the interpretation of the dream brings a succession of forms of interaction embedded in forms of interaction.

Within the model, in session 98, it becomes plausible that words are—or better said, become—the background for a figure of silence that, in the next move, turns around, so silences offer the background. In this complex figure-ground interplay, we witness “what meaning” is conveyed by “how such meaning is made.” For example, we witness how a “forgotten dream” is conveyed through “how silences are shaped” by the couple. A figure that is displayed on the canvas of words shows how Amalia and Thomä welcome a forgotten dream through the unsaid and arrive at interpreting it.

Silences or pauses play a role that is far from being semantically irrelevant. This interactional event moves beyond the view that silence is “the most powerful resistance” (Freud, 1912, p. 112) on the part of the patient, and “a powerful symptom” (Dimitrijević, 2021, p. 152) on the part of the analyst.

This role, observable in speaking and listening, leads to complex forms of interaction organization. In the 98th session, through the current model, a figure of silence enters the category of observables.

In the 98th session, while considering the analytic couple as a unit, we can see that words build up the canvas for *figures of silence*, and vice versa, as recursion builds up structure. We can also observe within such complex forms, in searching for a rhythmic expression, that the behavior of the analytic couple is not random. There is no pattern in chaos. Thus, we can say that in the 98th session, we encounter a particular *form of silence*—a form that preannounces content. Through this expectation of content, “forgetting” is surpassed; it manifests, and the analytic couple conveys a new semantic layer while creating it. In this session, we see that Amalia and Dr. Thomä are employing this kind of communication through how the silences are assembled into a jointly made foreground-background interplay.

Now the foreground is occupied by something unsaid, which eventually gives way to what is said. In this shift of what occupies the foreground and what slips into the background, a relationship between one form and another is achieved. We can observe this by comparing Figure 5 with Figure 6, where the same shape is achieved by the analytic couple through their interaction. Notably, reversing this foreground–background interplay also reverses the “form.”

## 7 Analytic couples and analytic thirds

From the “mind is inside” perspective, dyadic events or the emergence of a new level of relating (and thus of reality) provide few, if any, direct observables. The analytic third is one example. As meaning itself is not an “observable” but is the very substance from which distinctions are made in an environment, and through which reality and self are transformed, both “mind is inside” and “order” as systems of reference are needed. If correlated new facts can be observed, theories and models could eventually be put to the test.

If the analytic couple offers observable patterns, what makes this relevant for an analytic third? If such a process of making distinctions in the environment by the dyad is plausible within the present model, then the implications of this explicate order are well described by what would constitute an analytic third (Ogden, 1994; Benjamin, 2004). Such an (internal) object, shared by both, can be transformed from implicate to explicate, thus set into an observable field with proper tools. But this is not enough.

If there is an observable event, an explicit unfolding of actions, then the implicate of it, the analytic third in this case, calls for transformations if meaning-making is at work or being observed.

This new object requires a working through within the confines of the session, transforming the material by observing new facts, as well as adapting our own tools for making distinctions. Thus, we could arrive at making the implicate explicate. This new state of facts, if achievable, involves a new underlying organization. An explicate order always has an implicate order from which it emerges; in other words, there is no such thing as “something from nothing”, including “order out of chaos” transformed realities.

In our case, the path of rising complexity began from one direction, one dimension, which allowed for bidirectionality—“the analyst matters”—and has always had an implicate providing the means and patterns for an explicate, or an “unknown yet” providing an observational field emerging from it. For example, transference as explicate order had countertransference as implicate order in the Freudian beginnings of our field.

I believe that the core idea in “falsifiability” is the never-ending interplay of implicate into explicate order, of a new level yet to be observed. What follows is that bringing this new level of understanding into the explicate, through the analytic third (or other conceptualizations), will yield a new implicate order that informs our observations and distinctions in the new layer of reality we develop through our working through and our metaphors. Perhaps the analytic couple, which could provide observables in speaking and listening, can serve as an explicate for an implicate third. Viewed as a processual entity, the analytic couple needs to be part of the answer to “how order increases?” as variations in order entail process.

In the complex dynamic of interaction, it is conceivable to create patterns out of noise as a spontaneous emergence of structure. However, creating structure where none exists, while noise engenders a next level (e.g., transforming a sequence of nonsense into a meaningful narrative), is not possible. And here, in the 98th session, a pattern emerges, while neither Amalia nor Thomä can be qualified as the main authors of it.

## 8 Conclusion

The limitations of the current model, in fact a mapping device, are clear: the findings are still at the hypothesis-generating stage and imply inferences that require more empirical evidence.

The specificity of the model lies in using the session to code the session. The coding occurs under the operation “discrimination” applied to the set of autonomous elements of the session (e.g., sentences, turns, and narratives), resulting in a sequence of labels ready to receive meaning from the unfolding relationships. This reduces complexity while preserving the basic relations between elements and allows for a binary representation of the relationships among those relations. It should be noted that the coding is informed by CA practices; all similarities between one autonomous element and another are mainly CA-informed.

Is this sequence of forms obtained above potentially a result of a general organization of verbal interaction? Or does it stem from intrinsic properties of language use? Is it free from the contribution of the analytic frame that “enables an intersubjective exchange”, as well free from “the view of plausibility in the here and now”, as Thomä listens to Amalia? The answer is no. If we remember that mutuality, or a + b, which equates to level-stepping, emerges from the ongoing processes of meaning-making, then we should ask if context plays a part. There are many instances, outside the consulting room, where mutuality is achieved. Yet meaning-making is highly sensitive to context, whether outside or inside the consulting room. In considering supervision, interviewing, or teaching, mutuality occurs, of course. However, the analytic frame or the analytic attitude makes a difference. Explaining, clarifying, questioning, or interpreting something is fundamentally different from what occurs during interpretation within the session. “Mirror, mirror on the wall”, has no counterpart, as it gathers its meanings within the specific frame of analysis. It is an ‘interpretation’ arising from the interplay between the complex context created by Amalia and Thomä, Thomä’s understanding of transference (and countertransference), and the foundation upon which this context was constructed, the analytic frame.

The model is prone to evolve towards more complex and abstract formalizations, as the operation of discrimination (which employs the pair similar-different or operates within the fields of “order”) can be used to produce more complex tools (e.g., combinatorial algebras, Parker-Rhodes, 1981; Bastin et al., 1979).

For this specific analytic couple, Amalia and Dr. Thomä, while mirroring themselves, it is evident that distinctions are made by the analytic couple. There is potential to infer with more plausibility that there is a level of agency at work in what is defined as a third, which contributes to how change occurs.

How order is generated is open to observation through jointly achieved transformations, under “(A × B) → (A × B) (X)”. These transformations are neither random nor imposed by outside “forces” onto the analytic couple, nor by the patient alone or the analyst alone. It is an observable instance of self-organization of the couple under similarities and differences (and “similar differences and different similarities”).

What matters within the model is how speaking–listening gets organized through interaction. How Amalia’s utterances stand out against the backdrop of her own speech conjoined with Thomä’s listening, and how Thomä’s interpretation involves many layers of the here-and-now, while the background is also supplied by speaking–listening. A plausible hypothesis, under “change is based on lived experience” (Stern, 2004), would position authenticity, as an outcome of mutuality, as an important factor in how the analytic couple unfolds the making of meaning.

That listening governs the organization of verbal interaction is not a new idea, but the notion that speaking *and* listening provide the background for inscribing patterns is, and it seems that this can bring a new dimension within the field of analytic process research.

The analytic couple appears to *perform* a subtle transformation from the emotional experience of one participant to experiencing it collectively within the couple. This requires more research; yet in the 98th session, we witness how a ‘forgotten emotional experience’ (a dream) enters the transformation into a new emotional experience, evolving from silences to words, and eventually into a more adaptive form of interaction, such as knowing oneself through another. Within this context, we may hypothesize that there is an analytic third facilitating such triadic transformation.

The model serves as a formal theoretical instrument that reveals a new observational field that can be further developed. The exploratory nature of the model is evident, as is its dependence on interpretation, and the fact that it was applied in a single session within a single case (Amalia X) gives it a ‘demonstrative status’ pending further development.

It should be noted that inter-rater reliability has not yet been established, and replication across other cases or pathologies (or other ‘frames’ as well) needs to be addressed. However, this could set a trajectory toward the evaluation of hypotheses through empirical scrutiny. Nevertheless, the hypothesis could be developed further as it gains plausibility.

The findings support the idea that the analytic dyad plays a positive role in creating meaning by transforming noise into pattern, generating order out of chaos. They also pave the way for empirically researching the analytic third. Alongside the notion that ‘the analyst matters’, we can recognize that ‘the analytic couple matters’ as a source of pattern and meaning.

## Data Availability

The original contributions presented in the study are included in the article/supplementary material, further inquiries can be directed to the corresponding author.

## Footnotes

1 “A sentence is an abstract theoretical entity defined within a theory of grammar, while an utterance is the issuance of a sentence, a sentence-analogue, or sentence-fragment, in an actual context.” (Levinson, 1983 p. 18).

2 A process by which a structure or pattern emerges in the situation (i.e., analytic session) without specifications from the outside environment.

3 We use it every day, in what would be called a “clock arithmetic” (if binary then describes the day night cycle, if we choose a 12 or a 24 basis we arrive at a different cycle).

4 Akhtar (2007) underlines Thomä’s equanimity, his “respect for restraining and modulating the desire to interpret” (p. 697), and that under the understanding that “development is not once and for all; it is constant, ongoing, and often subtle” (p. 697). Commenting the material presented by Thomä at New Orleans (in 1994) Akhtar adds that “Dr. Thomä advances a working stance that accommodates discipline and spontaneity, tradition and freedom, and by implication, both the prose and the poetry of our clinical enterprise.” (p. 698).

5 In “Chaos: making a new science” Gleick (1987) quotes Robert Shaw as saying: “you don’t see something until you have the right metaphor to perceive it” (p. 262).

6 In “The German Specimen Case. Amalia X: Empirical Studies” (Kächele et al., 2006) we learn that Amalia X was born in 1939, and at 35 years old she begun a psychoanalytic treatment with Dr. Helmuth Thomä (517 sessions). The outcome was qualified as having good results. Amalia’s life story is documented in detail in many works (e.g., Levy et al., 2012; Thomä and Kächele, 1992). Thomä describes Amalia’s reason for analysis (Thomä and Kächele, 2007) as generated by “severe restrictions on her self-esteem” (Kächele et al., 2006, p.79), that entailed depression. Amalia suffered from the “severe strain resulting from her hirsutism”, that stigmatized her. On relational plan, other impediments to a rich relational life, according to Dr. Thomä, were due to compulsion neurosis and anxiety neurosis. She “was hard-working in her career, cultivated, single and quite feminine despite her stigma.” (Kächele et al., 2006, p.79).

## Author’s note

This article is based on a PhD thesis, “How the couple is observing the couple observing: A new model for qualitative process research”, developed under the supervision of Prof. Dr. Dr. Michael B. Buchholz (IPU Berlin) and Prof. Dr. Berndt Ahrbeck (Humboldt University Berlin). The use of clinical material from the 98th session of the German Specimen Case—Amalia X, including transcripts (thanks to Jörg Bergmann and Michael B. Buchholz) and recordings, has been granted by Prof. Dr. Anssi Peräkylä, Prof. Dr. Dr. Horst Kächele, and Prof. Dr. Dr. Michael B. Buchholz.
